# (*E*)-4-[2-(4-Eth­oxy­phen­yl)ethen­yl]-1-methyl­pyridinium naphthalene-2-sulfonate

**DOI:** 10.1107/S1600536813009240

**Published:** 2013-04-13

**Authors:** R. K. Balachandar, S. Kalainathan, P. G. Aravindan, Shibu M. Eappen, Jiban Podder

**Affiliations:** a Centre for Crystal Growth, School of Advanced Sciences, VIT University, Vellore 632 014, India; b Crystal Growth and Crystallography Division, School of Advanced Sciences, VIT University, Vellore 632 014, India; c Sophisticated Test and Instrumentation Centre (STIC), Cochin University PO, Cochin 682 022, Kerala, India; d Department of Physics, Bangladesh University of Engineering and Technology, Dhaka 1000, Bangladesh

## Abstract

In the title salt, C_16_H_18_NO^+^·C_10_H_7_O_3_S^−^, the substituents attached to the central C=C bond adopt a *trans* conformation and the benzene and pyridinium rings are nearly coplanar, making a dihedral angle of 6.01 (9)°. The crystal structure features weak C—H⋯O hydrogen bonds and C—H⋯π inter­actions .

## Related literature

The title compound was synthesized as part of a search for materials with non-linear optical properties, see: Okada *et al.* (1990 ▶); Yang *et al.* (2007 ▶). For the synthesis of the pyridinium precursor, see: Okada *et al.* (1990 ▶). For related compounds, see: Ruiz *et al.* (2006 ▶); Murugavel *et al.* (2009 ▶). 
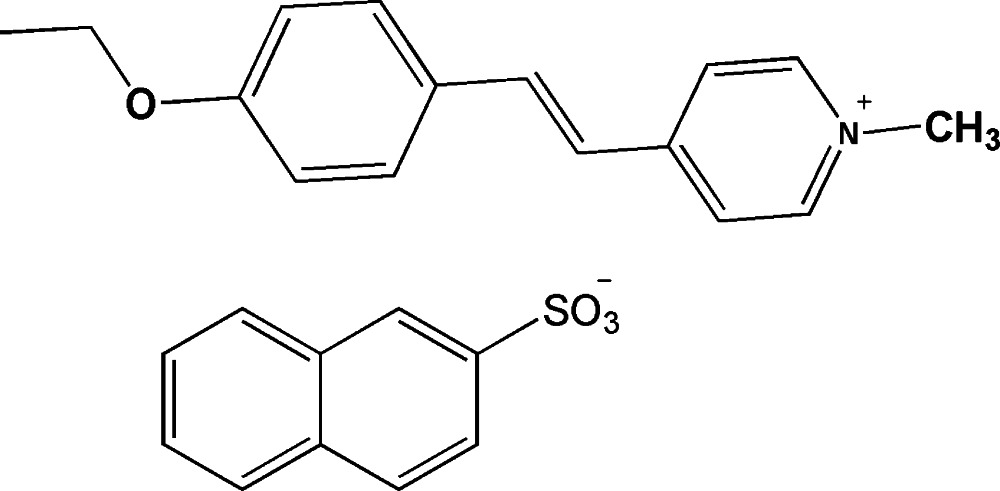


## Experimental

### 

#### Crystal data

C_16_H_18_NO^+^·C_10_H_7_O_3_S^−^
*M* *_r_* = 447.53
Monoclinic, 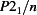

*a* = 10.896 (1) Å
*b* = 17.2838 (16) Å
*c* = 11.8888 (10) Å
β = 92.752 (4)°
*V* = 2236.4 (3) Å^3^
*Z* = 4
Mo *K*α radiation
μ = 0.18 mm^−1^
*T* = 296 K
0.40 × 0.35 × 0.30 mm

#### Data collection

Bruker APEXII CCD diffractometer
Absorption correction: multi-scan (*SADABS*; Bruker, 1999 ▶) *T*_min_ = 0.932, *T*_max_ = 0.949
9220 measured reflections
5354 independent reflections
3678 reflections with *I* > 2σ(*I*)
*R*_int_ = 0.020

#### Refinement

*R*[*F* ^2^ > 2σ(*F* ^2^)] = 0.052
*wR*(*F* ^2^) = 0.159
*S* = 1.03
5354 reflections
291 parameters
H-atom parameters constrained
Δρ_max_ = 0.52 e Å^−3^
Δρ_min_ = −0.32 e Å^−3^

### 

Data collection: *APEX2* (Bruker, 2004 ▶); cell refinement: *APEX2* and *SAINT* (Bruker, 2004 ▶); data reduction: *SAINT* and *XPREP* (Bruker, 2004 ▶); program(s) used to solve structure: *SIR92* (Altomare *et al.*, 1993 ▶); program(s) used to refine structure: *SHELXL97* (Sheldrick, 2008 ▶); molecular graphics: *ORTEP-3 for Windows* (Farrugia, 2012 ▶) and *Mercury* (Macrae *et al.*, 2006 ▶)’; software used to prepare material for publication: *SHELXL97*.

## Supplementary Material

Crystal structure: contains datablock(s) I, global. DOI: 10.1107/S1600536813009240/bh2474sup1.cif

Structure factors: contains datablock(s) I. DOI: 10.1107/S1600536813009240/bh2474Isup2.hkl

Supplementary material file. DOI: 10.1107/S1600536813009240/bh2474Isup3.cml

Additional supplementary materials:  crystallographic information; 3D view; checkCIF report

## Figures and Tables

**Table 1:** Hydrogen-bond geometry (Å, °) *Cg*1 is the centroid of the C20–C24 ring.

*D*—H⋯*A*	*D*—H	H⋯*A*	*D*⋯*A*	*D*—H⋯*A*
C10—H10⋯O3^i^	0.93	2.52	3.433 (3)	168
C11—H11⋯O3^ii^	0.93	2.42	3.323 (3)	165
C12—H12*B*⋯O4^ii^	0.96	2.58	3.488 (3)	159
C15—H15⋯O2^iii^	0.93	2.31	3.189 (3)	158
C25—H25⋯O3^iv^	0.93	2.45	3.323 (3)	156
C14—H14⋯*Cg*1^v^	0.93	2.84	3.686 (3)	152

Symmetry codes: (i) 
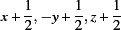
; (ii) 
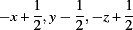
; (iii) 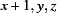; (iv) 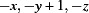; (v) 
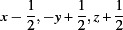
.

**Fig. 1.**
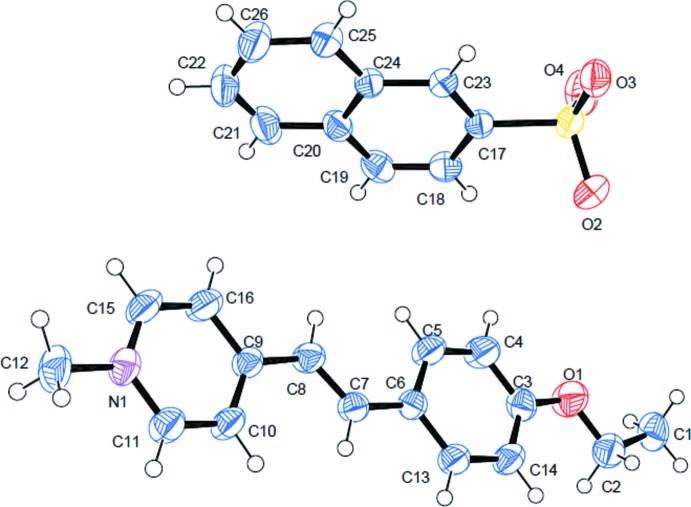
The molecular structure of the title compound, with 50% probability displacement ellipsoids.

**Fig. 2.**
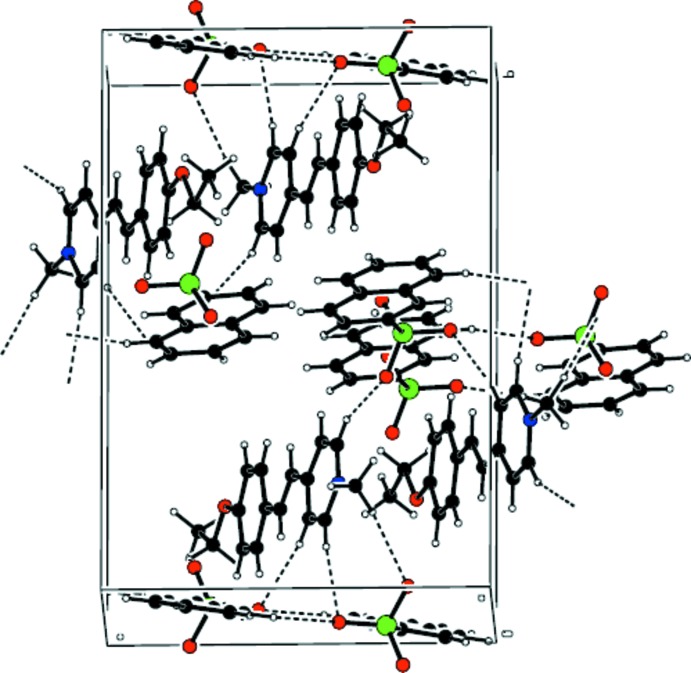
The crystal packing of the title salt, showing weak C—H···O, π···π aromatic and C—H···π interactions.

## supplementary crystallographic information

### Comment

The title compound was synthesized in the search for materials with non-linear optical properties (Okada *et al.*, 1990; Ruiz *et al.*, 2006; Yang *et al.*, 2007). In the title compound, C_16_H_18_NO^+^.C_10_H_7_O_2_S^-^, the pyridinium and benzene rings in the cation make a dihedral angle of 6.01 (9)°. This cation possess *trans* configuration, which can be confirmed from the torsion angle C6—C7—C8—C9, -177.8 (2)°. The C7═C8 group links the benzene and pyridinium rings, with a characteristic bond length of 1.329 (3) Å. These features are similar to those found in related compounds (Ruiz *et al.*, 2006; Murugavel *et al.*, 2009). All deviations from expected values for bond lengths are within *ca*. 0.05 Å. The ethoxy group has C1 and O1 atoms slightly deviated from the mean plane of the benzene ring, by 0.130 (2) and 0.015 (2) Å, respectively. The anion and cation are placed almost perpendicular each to other, the mean planes making an angle of 81.72 (6) Å.

Regarding the crystal packing, weak C—H···O hydrogen bonds and C—H···π interactions are stabilizing the crystal structure. The inter and intramolecular C—H···O interactions are formed mainly in cation-anion and anion-anion pairs. The pyridinium ring is significantly involved in the formation of C—H···O hydrogen bonds. Interestingly, there is a dimeric hydrogen bond between two symmetry-related anions (C25—H25···O3), and other hydrogen bonds exist between anions and cations (see Table 1). In addition, one C—H···π interaction is observed between the cation and the anion: C14—H14···*Cg^i^* (*Cg* is the centroid of ring C20/C21/C22/C24/C25/C26; symmetry code *i*: -1/2+*x*,1/2-*y*,1/2+*z*; see Table 1).

### Experimental

4-[2-(4-Ethoxy-phenyl)-vinyl-pyridinium iodide was obtained by condensation reaction between 1,4-dimethyl pyridinium iodide, which was prepared from 4-methylpyridine and methyl iodide, and 4-ethoxybenzaldehyde (all were taken in an equimolar ratio) in the presence of piperidine added as a catalyst. The solution was refluxed for 5 h, yielding the expected pyridinium salt after filtration (Okada *et al.*, 1990). Then the iodide salt was dissolved in water (20 ml) and aqueous sodium 2-naphthalenesulfonate was added. A yellow precipitate was formed, which was filtered off and dried in an oven at 413 K for 1 h (Ruiz *et al.*, 2006). Single crystals suitable for X-ray diffraction were obtained by successive recrystallization (three times) from a methanol/water (8:2 *v*/*v*) mixture.

### Refinement

H atoms were placed in calculated positions and allowed to ride on their parent atoms, with C—H distances in the range 0.93–0.97 Å and with *U*_iso_(H) = 1.2*U*_eq_(parent C) for CH and CH_2_ groups, and *U*_iso_(H) = 1.5*U*_eq_(parent C) for methyl groups.

### Crystal data

**Table:**

C_16_H_18_NO^+^·C_10_H_7_O_3_S^−^	*F*(000) = 944
*M**_r_* = 447.53	*D*_x_ = 1.329 Mg m^−^^3^
Monoclinic, *P*2_1_/*n*	Mo *K*α radiation, λ = 0.71073 Å
Hall symbol: -P 2yn	Cell parameters from 2935 reflections
*a* = 10.896 (1) Å	θ = 4.7–55.0°
*b* = 17.2838 (16) Å	µ = 0.18 mm^−^^1^
*c* = 11.8888 (10) Å	*T* = 296 K
β = 92.752 (4)°	Block, yellow
*V* = 2236.4 (3) Å^3^	0.40 × 0.35 × 0.30 mm
*Z* = 4	

### Data collection

**Table:**

Bruker APEXII CCD diffractometer	5354 independent reflections
Radiation source: fine-focus sealed tube	3678 reflections with *I* > 2σ(*I*)
Graphite monochromator	*R*_int_ = 0.020
ω and φ scan	θ_max_ = 28.3°, θ_min_ = 2.5°
Absorption correction: multi-scan (*SADABS*; Bruker, 1999)	*h* = −14→14
*T*_min_ = 0.932, *T*_max_ = 0.949	*k* = −22→11
9220 measured reflections	*l* = −15→9

### Refinement

**Table:**

Refinement on *F*^2^	Primary atom site location: structure-invariant direct methods
Least-squares matrix: full	Secondary atom site location: difference Fourier map
*R*[*F*^2^ > 2σ(*F*^2^)] = 0.052	Hydrogen site location: inferred from neighbouring sites
*wR*(*F*^2^) = 0.159	H-atom parameters constrained
*S* = 1.03	*w* = 1/[σ^2^(*F*_o_^2^) + (0.0795*P*)^2^ + 0.5684*P*] where *P* = (*F*_o_^2^ + 2*F*_c_^2^)/3
5354 reflections	(Δ/σ)_max_ = 0.004
291 parameters	Δρ_max_ = 0.52 e Å^−^^3^
0 restraints	Δρ_min_ = −0.32 e Å^−^^3^
0 constraints	

### Fractional atomic coordinates and isotropic or equivalent isotropic displacement parameters (Å^2^)

**Table:**

	*x*	*y*	*z*	*U*_iso_*/*U*_eq_	
S1	−0.13928 (4)	0.46834 (3)	0.22800 (4)	0.03758 (16)	
O1	−0.22890 (17)	0.26974 (11)	0.70188 (18)	0.0674 (5)	
O2	−0.17437 (15)	0.39399 (10)	0.27088 (15)	0.0621 (5)	
O3	−0.15214 (14)	0.47353 (9)	0.10620 (13)	0.0496 (4)	
O4	−0.19562 (15)	0.53257 (11)	0.28333 (15)	0.0614 (5)	
N1	0.67371 (16)	0.21961 (12)	0.39556 (15)	0.0439 (4)	
C1	−0.4210 (2)	0.24499 (16)	0.7732 (2)	0.0598 (7)	
H1A	−0.4018	0.2784	0.8361	0.090*	
H1B	−0.4758	0.2049	0.7956	0.090*	
H1C	−0.4596	0.2744	0.7129	0.090*	
C2	−0.3040 (2)	0.20899 (16)	0.7340 (2)	0.0569 (6)	
H2A	−0.2636	0.1794	0.7943	0.068*	
H2B	−0.3221	0.1747	0.6708	0.068*	
C3	−0.1151 (2)	0.25166 (14)	0.66518 (19)	0.0464 (5)	
C4	−0.0432 (2)	0.31443 (15)	0.6413 (2)	0.0565 (6)	
H4	−0.0741	0.3641	0.6501	0.068*	
C5	0.0732 (2)	0.30478 (14)	0.6047 (2)	0.0502 (6)	
H5	0.1200	0.3480	0.5885	0.060*	
C6	0.12266 (19)	0.23083 (13)	0.59137 (17)	0.0382 (5)	
C7	0.24500 (19)	0.21686 (13)	0.55280 (17)	0.0396 (5)	
H7	0.2698	0.1654	0.5500	0.048*	
C8	0.3257 (2)	0.26921 (13)	0.52101 (18)	0.0431 (5)	
H8	0.3042	0.3211	0.5255	0.052*	
C9	0.44579 (19)	0.25042 (12)	0.47955 (17)	0.0389 (5)	
C10	0.4916 (2)	0.17538 (13)	0.47388 (19)	0.0457 (5)	
H10	0.4452	0.1343	0.4992	0.055*	
C11	0.6037 (2)	0.16135 (14)	0.4317 (2)	0.0487 (5)	
H11	0.6322	0.1107	0.4279	0.058*	
C12	0.7940 (2)	0.20278 (17)	0.3504 (2)	0.0599 (7)	
H12A	0.8180	0.2449	0.3036	0.090*	
H12B	0.7888	0.1562	0.3066	0.090*	
H12C	0.8540	0.1963	0.4115	0.090*	
C13	0.0494 (2)	0.16808 (14)	0.6163 (2)	0.0469 (5)	
H13	0.0801	0.1183	0.6082	0.056*	
C14	−0.0689 (2)	0.17784 (14)	0.6530 (2)	0.0504 (6)	
H14	−0.1166	0.1350	0.6692	0.060*	
C15	0.6331 (2)	0.29222 (15)	0.4003 (2)	0.0551 (6)	
H15	0.6818	0.3323	0.3752	0.066*	
C16	0.5214 (2)	0.30866 (15)	0.4413 (2)	0.0559 (6)	
H16	0.4953	0.3598	0.4438	0.067*	
C17	0.02063 (18)	0.47725 (11)	0.26229 (16)	0.0335 (4)	
C18	0.0604 (2)	0.46691 (12)	0.37619 (17)	0.0403 (5)	
H18	0.0045	0.4519	0.4287	0.048*	
C19	0.1800 (2)	0.47885 (13)	0.40938 (18)	0.0457 (5)	
H19	0.2046	0.4733	0.4849	0.055*	
C20	0.26751 (19)	0.49955 (13)	0.33080 (19)	0.0417 (5)	
C21	0.3931 (2)	0.51132 (15)	0.3618 (2)	0.0567 (7)	
H21	0.4194	0.5074	0.4372	0.068*	
C22	0.4766 (2)	0.52831 (16)	0.2831 (3)	0.0651 (8)	
H22	0.5590	0.5348	0.3049	0.078*	
C23	0.10290 (18)	0.49640 (11)	0.18361 (16)	0.0341 (4)	
H23	0.0761	0.5022	0.1086	0.041*	
C24	0.22918 (18)	0.50750 (12)	0.21552 (17)	0.0367 (4)	
C25	0.3178 (2)	0.52578 (13)	0.1359 (2)	0.0476 (5)	
H25	0.2938	0.5309	0.0601	0.057*	
C26	0.4380 (2)	0.53582 (16)	0.1703 (3)	0.0620 (7)	
H26	0.4952	0.5479	0.1174	0.074*	

### Atomic displacement parameters (Å^2^)

**Table:**

	*U*^11^	*U*^22^	*U*^33^	*U*^12^	*U*^13^	*U*^23^
S1	0.0314 (3)	0.0373 (3)	0.0442 (3)	−0.0046 (2)	0.00312 (19)	−0.0015 (2)
O1	0.0487 (10)	0.0612 (12)	0.0944 (14)	0.0025 (9)	0.0244 (9)	0.0087 (10)
O2	0.0515 (10)	0.0550 (11)	0.0799 (12)	−0.0178 (8)	0.0034 (9)	0.0173 (9)
O3	0.0410 (8)	0.0584 (10)	0.0486 (9)	−0.0081 (7)	−0.0055 (6)	0.0012 (8)
O4	0.0419 (9)	0.0679 (12)	0.0748 (11)	0.0089 (8)	0.0046 (8)	−0.0213 (9)
N1	0.0359 (9)	0.0542 (12)	0.0424 (9)	−0.0050 (8)	0.0097 (7)	0.0004 (9)
C1	0.0369 (13)	0.0729 (19)	0.0707 (17)	0.0030 (12)	0.0144 (11)	−0.0028 (14)
C2	0.0491 (14)	0.0530 (16)	0.0687 (16)	−0.0018 (12)	0.0035 (12)	−0.0022 (13)
C3	0.0359 (11)	0.0528 (14)	0.0512 (13)	0.0054 (10)	0.0108 (9)	0.0038 (11)
C4	0.0502 (14)	0.0406 (13)	0.0805 (17)	0.0066 (11)	0.0215 (12)	0.0006 (12)
C5	0.0466 (13)	0.0386 (13)	0.0669 (15)	−0.0026 (10)	0.0168 (11)	0.0035 (11)
C6	0.0348 (10)	0.0409 (12)	0.0393 (10)	0.0007 (9)	0.0057 (8)	0.0026 (9)
C7	0.0403 (11)	0.0370 (11)	0.0419 (11)	0.0008 (9)	0.0057 (8)	0.0021 (9)
C8	0.0422 (12)	0.0369 (12)	0.0510 (12)	0.0015 (9)	0.0118 (9)	0.0027 (10)
C9	0.0380 (11)	0.0397 (12)	0.0395 (10)	−0.0029 (9)	0.0069 (8)	0.0030 (9)
C10	0.0423 (12)	0.0394 (12)	0.0568 (13)	−0.0086 (10)	0.0158 (10)	0.0021 (10)
C11	0.0465 (13)	0.0411 (13)	0.0597 (13)	−0.0033 (10)	0.0146 (10)	−0.0030 (11)
C12	0.0354 (12)	0.082 (2)	0.0635 (15)	−0.0020 (12)	0.0175 (10)	0.0022 (14)
C13	0.0407 (12)	0.0381 (12)	0.0628 (14)	0.0024 (10)	0.0105 (10)	0.0025 (11)
C14	0.0423 (12)	0.0450 (14)	0.0648 (14)	−0.0072 (10)	0.0114 (10)	0.0062 (11)
C15	0.0482 (14)	0.0476 (14)	0.0710 (16)	−0.0131 (11)	0.0179 (11)	0.0094 (12)
C16	0.0498 (14)	0.0390 (13)	0.0807 (17)	−0.0032 (11)	0.0209 (12)	0.0096 (12)
C17	0.0336 (10)	0.0283 (10)	0.0386 (10)	−0.0002 (8)	0.0004 (8)	−0.0022 (8)
C18	0.0456 (12)	0.0398 (12)	0.0358 (10)	0.0006 (9)	0.0050 (8)	0.0006 (9)
C19	0.0521 (13)	0.0468 (13)	0.0374 (10)	0.0054 (10)	−0.0070 (9)	−0.0005 (10)
C20	0.0391 (11)	0.0334 (11)	0.0517 (12)	0.0025 (9)	−0.0084 (9)	−0.0011 (10)
C21	0.0458 (13)	0.0534 (15)	0.0688 (15)	0.0015 (11)	−0.0197 (12)	−0.0015 (12)
C22	0.0343 (12)	0.0618 (17)	0.098 (2)	−0.0072 (12)	−0.0126 (13)	0.0035 (15)
C23	0.0341 (10)	0.0322 (10)	0.0359 (9)	−0.0023 (8)	0.0008 (7)	0.0015 (8)
C24	0.0341 (10)	0.0308 (10)	0.0449 (11)	−0.0008 (8)	−0.0004 (8)	0.0015 (9)
C25	0.0386 (12)	0.0463 (13)	0.0581 (13)	−0.0033 (10)	0.0036 (10)	0.0060 (11)
C26	0.0391 (13)	0.0583 (17)	0.089 (2)	−0.0074 (11)	0.0099 (12)	0.0050 (14)

### Geometric parameters (Å, º)

**Table:**

S1—O2	1.4408 (17)	C10—H10	0.9300
S1—O4	1.4425 (17)	C11—H11	0.9300
S1—O3	1.4510 (16)	C12—H12A	0.9600
S1—C17	1.777 (2)	C12—H12B	0.9600
O1—C3	1.371 (3)	C12—H12C	0.9600
O1—C2	1.396 (3)	C13—C14	1.390 (3)
N1—C15	1.333 (3)	C13—H13	0.9300
N1—C11	1.346 (3)	C14—H14	0.9300
N1—C12	1.470 (3)	C15—C16	1.362 (3)
C1—C2	1.512 (3)	C15—H15	0.9300
C1—H1A	0.9600	C16—H16	0.9300
C1—H1B	0.9600	C17—C23	1.367 (3)
C1—H1C	0.9600	C17—C18	1.413 (3)
C2—H2A	0.9700	C18—C19	1.359 (3)
C2—H2B	0.9700	C18—H18	0.9300
C3—C4	1.375 (3)	C19—C20	1.412 (3)
C3—C14	1.382 (3)	C19—H19	0.9300
C4—C5	1.371 (3)	C20—C21	1.415 (3)
C4—H4	0.9300	C20—C24	1.420 (3)
C5—C6	1.399 (3)	C21—C22	1.368 (4)
C5—H5	0.9300	C21—H21	0.9300
C6—C13	1.387 (3)	C22—C26	1.392 (4)
C6—C7	1.451 (3)	C22—H22	0.9300
C7—C8	1.329 (3)	C23—C24	1.423 (3)
C7—H7	0.9300	C23—H23	0.9300
C8—C9	1.457 (3)	C24—C25	1.420 (3)
C8—H8	0.9300	C25—C26	1.365 (3)
C9—C16	1.391 (3)	C25—H25	0.9300
C9—C10	1.393 (3)	C26—H26	0.9300
C10—C11	1.364 (3)		
			
O2—S1—O4	113.52 (11)	N1—C12—H12A	109.5
O2—S1—O3	113.19 (10)	N1—C12—H12B	109.5
O4—S1—O3	112.62 (11)	H12A—C12—H12B	109.5
O2—S1—C17	105.67 (10)	N1—C12—H12C	109.5
O4—S1—C17	105.23 (10)	H12A—C12—H12C	109.5
O3—S1—C17	105.68 (9)	H12B—C12—H12C	109.5
C3—O1—C2	117.9 (2)	C6—C13—C14	121.6 (2)
C15—N1—C11	119.78 (19)	C6—C13—H13	119.2
C15—N1—C12	120.4 (2)	C14—C13—H13	119.2
C11—N1—C12	119.9 (2)	C3—C14—C13	119.5 (2)
C2—C1—H1A	109.5	C3—C14—H14	120.2
C2—C1—H1B	109.5	C13—C14—H14	120.2
H1A—C1—H1B	109.5	N1—C15—C16	121.0 (2)
C2—C1—H1C	109.5	N1—C15—H15	119.5
H1A—C1—H1C	109.5	C16—C15—H15	119.5
H1B—C1—H1C	109.5	C15—C16—C9	121.3 (2)
O1—C2—C1	106.8 (2)	C15—C16—H16	119.4
O1—C2—H2A	110.4	C9—C16—H16	119.4
C1—C2—H2A	110.4	C23—C17—C18	120.35 (18)
O1—C2—H2B	110.4	C23—C17—S1	122.14 (15)
C1—C2—H2B	110.4	C18—C17—S1	117.46 (15)
H2A—C2—H2B	108.6	C19—C18—C17	120.31 (19)
O1—C3—C4	114.7 (2)	C19—C18—H18	119.8
O1—C3—C14	125.7 (2)	C17—C18—H18	119.8
C4—C3—C14	119.5 (2)	C18—C19—C20	120.97 (19)
C5—C4—C3	120.9 (2)	C18—C19—H19	119.5
C5—C4—H4	119.5	C20—C19—H19	119.5
C3—C4—H4	119.5	C19—C20—C21	122.6 (2)
C4—C5—C6	121.0 (2)	C19—C20—C24	119.10 (18)
C4—C5—H5	119.5	C21—C20—C24	118.3 (2)
C6—C5—H5	119.5	C22—C21—C20	121.3 (2)
C13—C6—C5	117.5 (2)	C22—C21—H21	119.4
C13—C6—C7	119.0 (2)	C20—C21—H21	119.4
C5—C6—C7	123.6 (2)	C21—C22—C26	120.0 (2)
C8—C7—C6	127.4 (2)	C21—C22—H22	120.0
C8—C7—H7	116.3	C26—C22—H22	120.0
C6—C7—H7	116.3	C17—C23—C24	120.52 (17)
C7—C8—C9	124.2 (2)	C17—C23—H23	119.7
C7—C8—H8	117.9	C24—C23—H23	119.7
C9—C8—H8	117.9	C25—C24—C20	119.16 (19)
C16—C9—C10	116.0 (2)	C25—C24—C23	122.15 (18)
C16—C9—C8	120.3 (2)	C20—C24—C23	118.69 (18)
C10—C9—C8	123.65 (19)	C26—C25—C24	120.2 (2)
C11—C10—C9	120.9 (2)	C26—C25—H25	119.9
C11—C10—H10	119.6	C24—C25—H25	119.9
C9—C10—H10	119.6	C25—C26—C22	121.2 (2)
N1—C11—C10	121.0 (2)	C25—C26—H26	119.4
N1—C11—H11	119.5	C22—C26—H26	119.4
C10—C11—H11	119.5		

### Hydrogen-bond geometry (Å, º)

Cg1 is the centroid of the C20–C24 ring.

**Table:**

*D*—H···*A*	*D*—H	H···*A*	*D*···*A*	*D*—H···*A*
C23—H23···O3	0.93	2.54	2.912 (2)	105
C10—H10···O3^i^	0.93	2.52	3.433 (3)	168
C11—H11···O3^ii^	0.93	2.42	3.323 (3)	165
C12—H12*B*···O4^ii^	0.96	2.58	3.488 (3)	159
C15—H15···O2^iii^	0.93	2.31	3.189 (3)	158
C25—H25···O3^iv^	0.93	2.45	3.323 (3)	156
C14—H14···*Cg*1^v^	0.93	2.84	3.686 (3)	152

Symmetry codes: (i) *x*+1/2, −*y*+1/2, *z*+1/2; (ii) −*x*+1/2, *y*−1/2, −*z*+1/2; (iii) *x*+1, *y*, *z*; (iv) −*x*, −*y*+1, −*z*; (v) *x*−1/2, −*y*+1/2, *z*+1/2.

## References

[bb1] Altomare, A., Cascarano, G., Giacovazzo, C. & Guagliardi, A. (1993). *J. Appl. Cryst.* **26**, 343–350.

[bb2] Bruker (1999). *SADABS* Bruker AXS Inc., Madison, Wisconsin, USA.

[bb3] Bruker (2004). *APEX2*, *SAINT* and *XPREP* Bruker AXS Inc., Madison, Wisconsin, USA.

[bb4] Farrugia, L. J. (2012). *J. Appl. Cryst.* **45**, 849–854.

[bb5] Macrae, C. F., Edgington, P. R., McCabe, P., Pidcock, E., Shields, G. P., Taylor, R., Towler, M. & van de Streek, J. (2006). *J. Appl. Cryst.* **39**, 453–457.

[bb6] Murugavel, S., SubbiahPandi, A., Srikanth, C. & Kalainathan, S. (2009). *Acta Cryst.* E**65**, o71. 10.1107/S1600536808041007 PMC2967981 21581710

[bb7] Okada, S., Masaki, A., Matsuda, H., Nakanishi, H., Kato, M., Muramatsu, R. & Otsuka, M. (1990). *Jpn. J. Appl. Phys* **29**, 1112–1115.

[bb8] Ruiz, B., Yang, Z., Gramlich, V., Jazbinsek, M. & Günter, P. (2006). *J. Mater. Chem* **16**, 2839–2842.

[bb9] Sheldrick, G. M. (2008). *Acta Cryst.* A**64**, 112–122. 10.1107/S0108767307043930 18156677

[bb10] Yang, Z., Jazbinsek, M., Ruiz, B., Aravazhi, S., Gramlich, V. & Günter, P. (2007). *Chem. Mater* **19**, 3512–3518.

